# Toward uncertainty-aware clinical decision support for treatment response prediction in metastatic NSCLC: integrating FDG-PET, T-cell repertoire, and cytokines with conformal prediction

**DOI:** 10.21203/rs.3.rs-10681426/v1

**Published:** 2026-08-27

**Authors:** Faisal Yaseen, Daniel S. Hippe, Sunan Cui, Jie Fu, Yejin Kim, Clemens Grassberger, Lei Deng, Ting Ye, Paul E Kinahan, Jing Zeng, John H Gennari, Stephen R. Bowen

**Affiliations:** University of Washington; Fred Hutch Cancer Center: Fred Hutchinson Cancer Center; University of Washington; University of Washington; University of Washington; University of Washington; University of Washington; University of Washington; University of Washington; University of Washington; University of Washington; University of Washington

**Keywords:** Clinical decision support systems, Conformal prediction, Uncertainty quantification, Multimodal machine learning, FDG-PET, T-cell receptor repertoire, Cytokines, Non-small cell lung cancer, Immunotherapy, Treatment response prediction

## Abstract

**Background:**

Variable response to chemoimmunotherapy in metastatic non-small cell lung cancer (mNSCLC), together with the limited discriminative accuracy of PD-L1 tumor proportion score, highlights the need for reliable, uncertainty-aware early response prediction using multimodal biomarkers. We developed and prototyped a multimodal clinical decision support (CDS) framework integrating longitudinal FDG-PET, T-cell receptor (TCR), and cytokine biomarkers with conformal prediction to deliver uncertainty-quantified, patient-level response predictions.

**Methods:**

Thirty-five patients with mNSCLC receiving first-line carboplatin–pemetrexed–pembrolizumab on the PET-BRIGHT trial (NCT04151940) underwent FDG-PET/CT and blood collection at baseline and week 3. Multimodal biomarkers included FDG-PET metrics (standardized uptake value/total lesion glycolysis), TCR diversity metrics, and inflammatory cytokines. Nested leave-one-out cross-validation with automated feature selection identified a single biomarker per modality. Class-balanced logistic regression was used for classification, with discrimination assessed by the area under the receiver operating characteristic curve (AUROC) and calibration by the Brier score. Conformal prediction (targeting 80% reliability) quantified patient-level uncertainty via prediction sets and singleton rate. Unimodal and multimodal early- and late-fusion models were evaluated using baseline-only and combined baseline plus mid-treatment biomarkers. Models were benchmarked against PD-L1 tumor proportion score, and statistical significance was assessed by permutation testing against an empirical null.

**Results:**

PD-L1 tumor proportion score, the current clinical standard, discriminated poorly (AUROC 0.58, 95% CI 0.39–0.78). At PreTx, unimodal TCR was the strongest single modality (AUROC 0.80), followed by PET (0.76) and cytokines (0.54). Late fusion of PET and TCR achieved the highest PreTx discrimination (0.85) and increased singleton predictions from 78% to 89%. After incorporating mid-treatment biomarkers, cytokines became the strongest single modality (0.78) while TCR was attenuated (0.66); late fusion of PET and cytokines achieved the highest discrimination overall (0.86). Thirteen of 22 model and timepoint combinations exceeded a permutation null at p < 0.05, and empirical coverage was 78% and 82% against an 80% target.

**Conclusions:**

A multimodal framework combining longitudinal biomarkers with conformal prediction substantially outperformed the current clinical standard while identifying patients for whom a confident prediction could not be made. A prototype CDS interface demonstrates feasibility for clinical translation.

**Trial registration:**

ClinicalTrials.gov NCT04151940, registered 26 September 2019.

## Background

Patients with metastatic non-small cell lung cancer (mNSCLC) without targetable genomic alterations experience highly variable responses to first-line chemoimmunotherapy[1, 2]. Although immune checkpoint inhibitors combined with chemotherapy (chemoICI) have improved survival outcomes for some patients, nearly half of all treated patients (objective response rate ~ 45–55%) derive limited clinical benefit while still experiencing treatment-related toxicity and financial burden[2, 3]. This heterogeneity in treatment response and outcomes creates a critical unmet need for early response assessments that can inform treatment escalation, de-escalation, consolidation, or modification within the first few weeks of therapy to support more personalized treatment strategies.

The most widely used clinical biomarkers, including programmed death ligand 1 (PD-L1) and tumor mutation burden (TMB), have limited predictive performance in guiding treatment response in metastatic NSCLC. Clinical benefit has been observed across varying levels of PD-L1[4-6]. PD-L1 nonetheless remains the biomarker most widely used to guide treatment decisions in this setting. Similarly, TMB has also shown variable predictive value, with differences in measurement methods and inconsistent associations with clinical outcomes limiting its routine clinical use[6, 7]. Furthermore, single-modality biomarkers cannot fully capture the complex interaction between tumor burden, immune response, and systemic inflammation that influences the treatment response. These limitations highlight the need for an integrated multimodal approach to improve prediction accuracy and personalized treatment strategies[8-10].

Non-invasive molecular imaging and circulating immune biomarkers provide complementary insights into tumor characteristics and host immune response. Quantitative measurement of 18F-fluorodeoxyglucose uptake by positron emission tomography (FDG-PET) characterizes metabolic tumor activity and has demonstrated value for early response assessment during systemic therapy[11-13]. Circulating immune biomarkers including T cell receptor (TCR) repertoire metrics and inflammatory cytokines provide information about systemic immune response dynamics that cannot be captured by imaging alone[14-16]. Prior studies in mNSCLC have shown that integration of multiple data modalities improves treatment response prediction compared with unimodal approaches [17, 18]. These findings support the potential of multimodal and longitudinal biomarker strategies to improve early treatment response prediction.

Translating predictive models into clinical decision support systems (CDSS) raises requirements beyond discriminative accuracy. A CDSS must convey not only what a model predicts but how much confidence that prediction warrants for the individual patient in front of the clinician. Accurate probability estimation, and the joint optimization of calibration alongside discrimination, have been identified as central unresolved methodological challenges for the development of data-driven clinical decision processes[19, 20]. This gap is consequential in oncology, where a single early prediction may influence whether therapy is continued, intensified, or abandoned. Multimodal models tend to produce poorly calibrated probability estimates, so that apparent gains in discrimination do not necessarily translate into predictions a clinician can act upon. A model that cannot signal when it should not be trusted provides limited support for clinical reasoning, regardless of its aggregate performance.

Conformal prediction addresses this limitation by attaching an explicit, patient-specific measure of confidence to every prediction. Unlike Bayesian or bootstrap-based approaches that require distributional assumptions, conformal prediction is distribution-free and model-agnostic and provides finite-sample validity guarantees that hold for any sample size[21]. Rather than forcing a binary label, it returns a prediction set that contains the true class with a user-specified probability. When the model is confident the set contains a single class; when the available evidence is genuinely ambiguous the set contains both, and the case is flagged rather than resolved arbitrarily. An abstention is therefore informative in its own right: it marks the patients for whom further evidence should be sought before a treatment decision is made.

In this study, we develop and evaluate an uncertainty-aware multimodal framework for early response prediction in mNSCLC, using synchronized FDG-PET, TCR repertoire, and cytokine biomarkers collected prospectively at baseline and after one cycle of chemoICI. This work had three objectives: (1) to determine whether multimodal fusion improves discrimination and probability calibration relative to unimodal models and to PD-L1; (2) to quantify patient-level uncertainty using conformal prediction and to characterize the resulting trade-off between decisiveness and reliability; and (3) to demonstrate feasibility of clinical translation by implementing the framework as a functioning decision support prototype. To our knowledge, this is among the first prospective studies to integrate longitudinal FDG-PET imaging with circulating immune biomarkers under a conformal prediction framework and to implement the resulting model as a clinical decision support prototype.

## Methods

### Study design and participants

Participants were enrolled in PET-BRIGHT (NCT04151940), a prospective clinical trial approved by the Institutional Review Board (IRB #10203). Eligible participants had histologically or cytologically confirmed stage IV mNSCLC (AJCC v8), Eastern Cooperative Oncology Group performance status 0–2, at least one measurable lesion by RECIST v1.1, and no prior systemic therapy for advanced disease. All participants provided written informed consent. Between November 2019 and November 2023, 35 participants were enrolled and received first-line platinum-doublet chemotherapy combined with pembrolizumab (carboplatin–pemetrexed for non-squamous histology, n = 30; carboplatin–paclitaxel for squamous histology, n = 5).

Synchronous FDG-PET/CT imaging and peripheral blood sampling were performed per trial protocol before treatment initiation (PreTx) and at week 3, following one cycle of chemoimmunotherapy (MidTx). Of 35 enrolled participants, 27 were evaluable for response at week 12. Eight were not evaluable owing to severe adverse events following the first treatment cycle, withdrawal of consent), or loss to follow-up.

### Response assessment

Tumor response was assessed at week 12 using RECIST v1.1, after four cycles of chemoimmunotherapy. Participants achieving complete or partial response were classified as responders; those with stable or progressive disease were classified as non-responders. For binary classification, responders were encoded as class 0 and non-responders as class 1.

### Multimodal biomarker acquisition

#### FDG-PET imaging

FDG PET/CT scans were acquired on a GE Discovery MI scanner at 60 minutes post-injection, 2.5 minutes per bed position at baseline (PreTx) and mid-treatment (MidTx). Primary metabolic tumor volume (MTV) was delineated using semi-automated gradient-based segmentation (PET Edge, MIM Software) with consensus from a board-certified multidisciplinary team (medical physicist, medical/radiation oncologist). Images were rigidly co-registered around the primary MTV. Standardized uptake values (SUVmax, SUVpeak) and total lesion glycolysis (TLG, the product of SUVmean and MTV) were extracted from the primary MTV and, separately, aggregated across lung and liver lesions.

#### T-cell receptor repertoire

TCR β-chain sequencing was performed on peripheral blood mononuclear cell genomic DNA (immunoSEQ, Adaptive Biotechnologies) at a survey sampling depth of 120,000 T cells. Repertoire metrics comprised diversity/evenness (Pielou evenness), richness (total productive templates, productive rearrangements), and clonality (Simpson clonality).

#### Cytokine profiling

Fourteen circulating cytokines spanning pro-inflammatory, anti-inflammatory, Th2/immunomodulatory, T-cell growth, and chemokine classes were quantified using multiplex immunoassay (IsoCode Human Adaptive Immune chips, IsoPlexis) and ELISA. Values below the limit of detection were replaced with half the limit of detection.

The multimodal biomarkers including PET, TCR and cytokines considered are established biomarkers supported by prior literature[22-25]. The multimodal biomarkers used in the analysis are shown in the Supplementary Table 1.

### Model development and cross-validation

Features were log (1 + x) transformed after clipping negative values to zero, to reduce the right skew characteristic of SUV, template count, and cytokine distributions, then mean-imputed and standardized to zero mean and unit variance. All preprocessing parameters, including imputation means and standardization coefficients, were estimated within each training fold and applied unchanged to the held-out participant, preventing information leakage. PET measurements were complete for all evaluable participants; missing values affected 5.4% of measurements in the PreTx analysis and 4.9% in the combined analysis (Supplementary Table 2).

Analyses were performed in two temporal settings: a baseline model using PreTx features alone, representing the therapy-selection decision point, and a combined model additionally incorporating MidTx features, representing the potential adaptation decision point at week 3. Model development used nested leave-one-out cross-validation (LOOCV). In each of the 27 outer folds, one participant was held out and the remaining 26 formed the training set. Within the training set, an inner LOOCV loop evaluated every candidate feature within each modality individually, and the feature achieving the highest AUROC was selected as that modality's representative. Restricting each modality to a single feature maintained a favorable events-per-predictor ratio while preserving modality-specific information. The selected features were then used to fit the outer-fold model, which was applied to the held-out participant; no feature selection was performed using the full dataset.

Classification used logistic regression with class weights inversely proportional to class frequency to account for the 2:1 outcome imbalance. Logistic regression was chosen because it produces well-calibrated probability estimates suitable for conformal calibration. Models were fitted without regularization; an L2 penalty (C = 1.0) was applied as a fallback in any fold where the unpenalized fit failed to converge. Feature-selection stability was quantified as the proportion of outer folds in which each feature was selected.

Two fusion strategies were compared. In early fusion, the selected features from each modality were concatenated into a single design matrix and a single logistic regression model was fitted. In late fusion, independent modality-specific models were fitted and their predicted probabilities averaged with equal weights to yield the combined prediction. All pairwise and three-way modality combinations were evaluated under both strategies, giving 11 models per temporal setting: three unimodal, six bimodal (three modality pairs under each fusion strategy), and two trimodal.

### Conformal prediction

Conformal prediction was applied to convert model probabilities into prediction sets with finite-sample validity. Because the cohort size precluded reserving a dedicated calibration split, we used a jackknife+ construction in which out-of-fold predictions were generated by leave-one-out refitting within each outer-fold training set [26]. Nonconformity scores were defined as one minus the predicted probability of the class under consideration, so that higher scores indicate greater disagreement between model and observation. For a target error rate α, the threshold was the ⌈(n+1)(1-α)⌉/n empirical quantile of the calibration scores. A class label was included in the prediction set for a held-out participant if its nonconformity score fell at or below this threshold. The resulting set could contain a single label (responder {0} or non-responder {1}) or both labels ({0,1}) when the evidence was insufficient to exclude either. Empty sets, which arise when neither label meets the threshold, were assigned both labels by convention so that every participant received either a decisive or an explicitly ambiguous prediction. A target coverage of 80% (α = 0.20) was pre-specified. This level balances reliability against conformal efficiency: higher coverage targets (90–95%) would substantially increase the proportion of ambiguous prediction sets in a cohort of this size, reducing the proportion of participants receiving an actionable single-class prediction.

### Statistical analysis

Categorical variables are summarized as counts and percentages, and continuous variables as median and range. Model discrimination was quantified using the area under the receiver operating characteristic curve (AUROC), with responders treated as the positive class. Probabilistic calibration was assessed using the Brier score, which is the mean squared difference between predicted probabilities and observed binary outcomes, with lower values indicating closer agreement[27,28]. Ninety-five percent confidence intervals for all performance metrics were obtained by percentile bootstrap using 1,000 resamples of the pooled cross-validated predictions[29].

Performance of the conformal prediction layer was summarized by three quantities. Empirical coverage is the proportion of participants whose prediction set contained the observed outcome, and measures reliability against the pre-specified 80% target. Mean prediction set size and singleton rate, defined as the proportion of participants receiving a single-class prediction, measure decisiveness. Coverage and singleton rate are distinct and complementary: a model may achieve valid coverage while returning many ambiguous sets, and conformal prediction trades reliability against decisiveness as the target error rate is varied [30,31]. Both are therefore reported for every model.

Model performance was benchmarked against PD-L1 tumor proportion score dichotomized at 1%, the biomarker used in routine practice to inform first-line treatment decisions. Because PD-L1 is a directly measured clinical assay rather than a fitted model, no parameters are estimated and cross-validation is not applicable; its performance was therefore evaluated directly on the evaluable cohort. This yields an in-sample estimate for the comparator and a cross-validated estimate for the multimodal models, a comparison that is conservative with respect to the models under evaluation.

Given the cohort size, statistical significance was assessed primarily by permutation testing against an empirical null distribution. Response labels were randomly permuted, and the complete nested leave-one-out cross-validation pipeline, including inner-loop feature selection, preprocessing, model fitting, and probability generation, was re-executed in full on each permuted dataset. This procedure was repeated 500 times at each temporal setting. The empirical p-value was calculated as the number of permuted AUROC values greater than or equal to the observed value, plus one, divided by the number of permutations plus one [32,33]. Because the null distribution is generated by applying the identical analytic procedure to data in which any association between biomarkers and outcome has been destroyed, this approach accounts for optimism introduced by feature selection and by the resampling scheme itself, and provides a reference distribution appropriate to the specific pipeline rather than to an idealized model.

Paired DeLong tests comparing AUROC between unimodal and multimodal models are reported secondarily, as effect estimates with confidence intervals rather than as significance tests, because pairwise comparison of correlated models is underpowered at this sample size [34]. Leave-one-out cross-validation was pre-specified as the primary resampling scheme in order to maximize training set size. Because pooled leave-one-out AUROC estimation may be biased in small samples [35,36], repeated stratified five-fold cross-validation with 100 repetitions was performed as a sensitivity analysis, and results from both schemes are reported. Two additional analyses characterized framework behavior at the level of individual participants. Reclassification was quantified as the number of participants whose prediction changed from incorrect to correct, or from ambiguous to correct, following the addition of each modality. The relationship between decisiveness and reliability was characterized by comparing classification accuracy among participants receiving singleton predictions with accuracy across the full cohort.

All analyses were performed in Python version 3.10 using scikit-learn version 1.7.2, NumPy, pandas, and SciPy. All stochastic procedures used a fixed random seed. Permutation p values are one-sided by construction; DeLong comparisons are two-sided. P values below 0.05 were considered statistically significant.

## Results

### Patient characteristics

The study cohort included 35 patients with mNSCLC (median age, 71 years; range, 33–84). Comprehensive demographic and clinical characteristics for the full cohort are presented in Table 1. Among the evaluable population, 18 patients (66.7%) were responders (class 0), while 9 patients (33.3%) were non-responders (class 1).

### Performance of clinical benchmark

PD-L1 expression discriminated poorly between responders and non-responders. The response rate was 75.0% among participants with PD-L1 >1% (9 of 12) and 60.0% among those with PD-L1 <1% (9 of 15) (Supplementary Table 3). This corresponded to an AUROC of 0.583 (95% CI 0.388–0.782), with sensitivity 0.500 and specificity 0.667. The confidence interval includes 0.5, indicating that PD-L1 alone was not distinguishable from chance discrimination in this cohort.

### Feature selection stability

Selection frequencies for each candidate feature are shown in Supplementary Fig 1. Imaging selection was invariant: MTV SUVpeak measured before treatment was selected as the PET representative in every fold, in both the baseline and combined analyses. Blood biomarker selection was stable at baseline for TCR (Pielou evenness, 26 of 27 folds) but unstable for cytokines, where TGF-β1 was selected in 16 folds, IL-6 in 10, and MIP-1β in 1. When mid-treatment measurements were included, cytokine selection became highly stable (IL-6 at week 3, 26 of 27 folds) while TCR selection divided predominantly between baseline and mid-treatment Pielou evenness (16 and 9 folds respectively). The instability of baseline cytokine selection is consistent with the weak discriminative performance of that model reported below.

### Predictive Performance of Unimodal and Multimodal Models Across Timepoints

#### Baseline predictive performance

**Figure 2:**
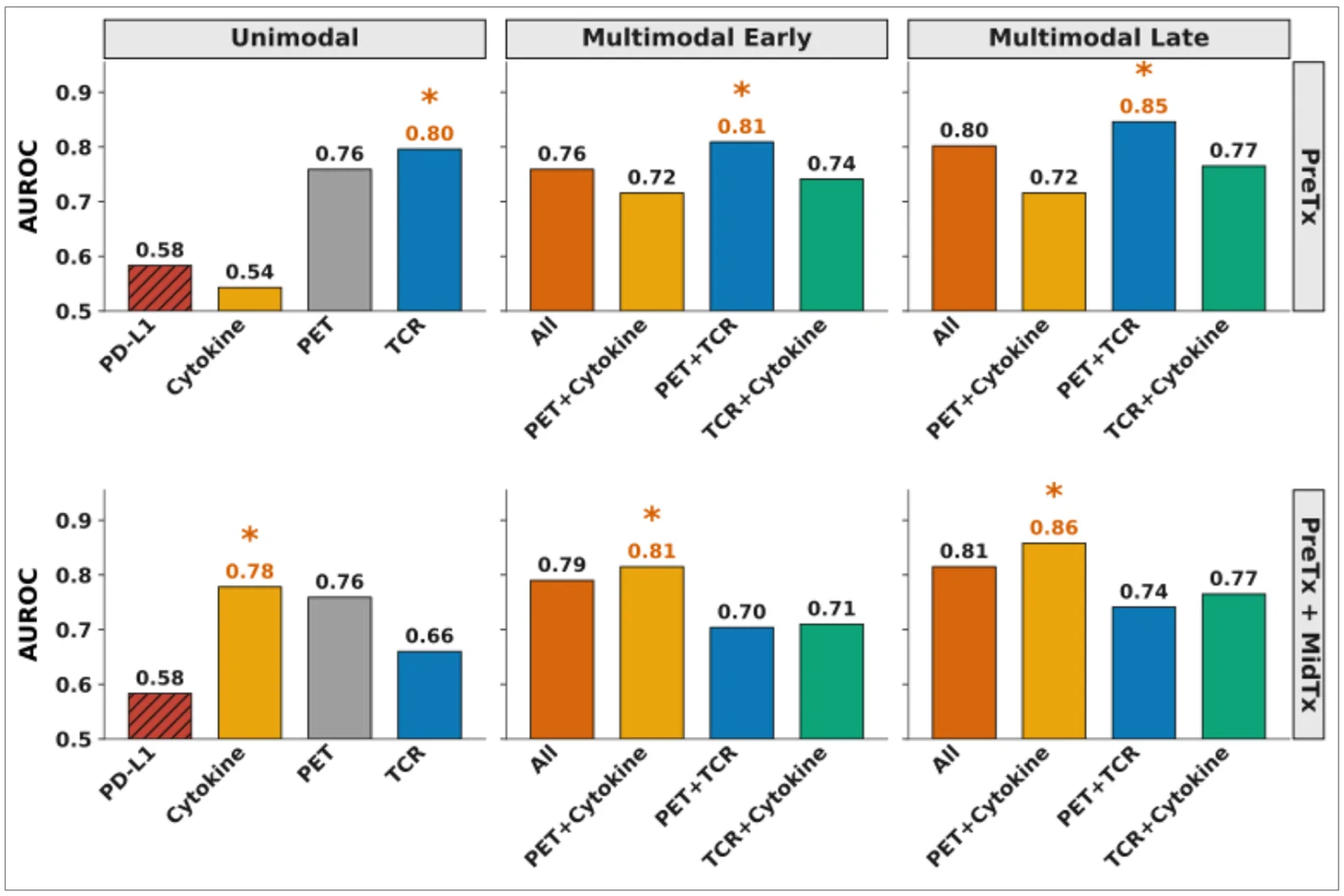
Discriminative performance of unimodal and multimodal models at both temporal settings, referenced to the current clinical standard. Bars show the area under the receiver operating characteristic curve (AUROC) derived from pooled predictions across 27 nested leave-one-out cross-validation folds, with responders as the positive class. Columns group models by fusion strategy: unimodal, early fusion (selected features concatenated before modelling), and late fusion (modality-specific predicted probabilities averaged). Rows show models developed using pre-treatment biomarkers alone (PreTx) and using pre- and mid-treatment biomarkers combined (PreTx + MidTx). PD-L1 tumor proportion score dichotomized at the 1% threshold (red, hatched) is shown as the first bar of each unimodal panel for reference; PD-L1 is a single pre-treatment measurement and therefore does not differ between rows. Asterisks and orange values denote the highest-performing model within each panel and do not indicate statistical significance; 95% confidence intervals and permutation-based p-values for all models are reported in Supplementary Tables 4-6. *All*, PET + TCR + cytokine.

**Figure 3:**
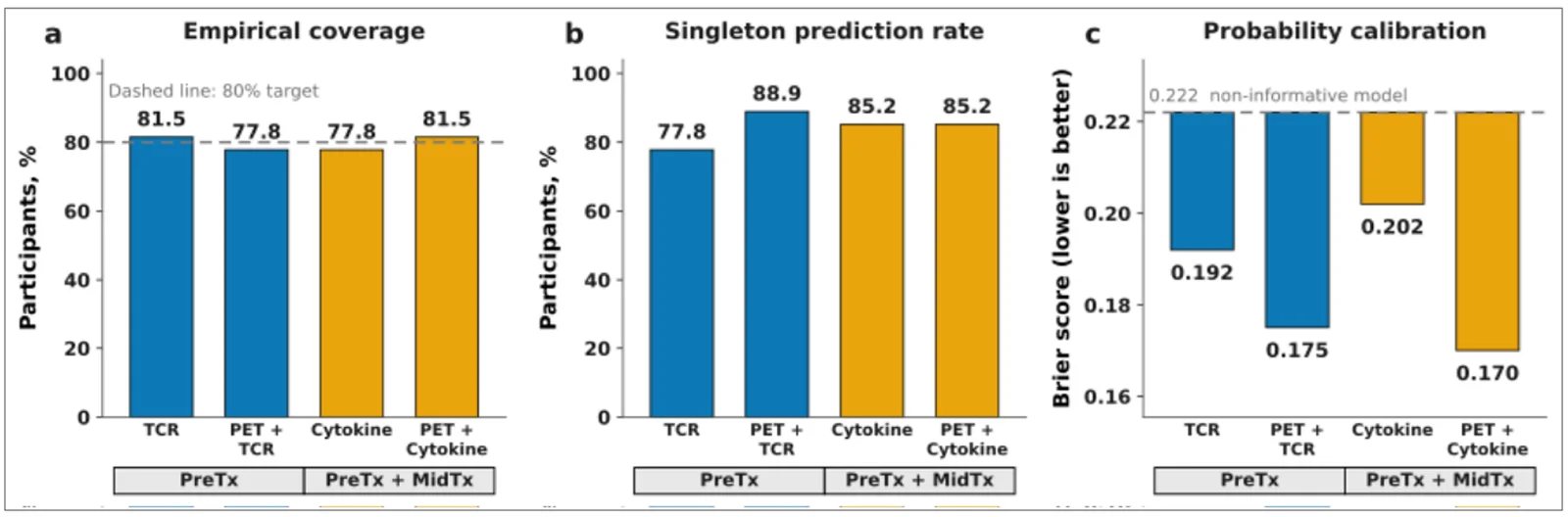
Prediction confidence and calibration across selected models. **(a)**Empirical coverage, the proportion of participants whose prediction set contained the observed outcome; the dashed line marks the 80% target (α = 0.20). **(b)**Singleton prediction rate, the proportion of participants receiving a single-class rather than an ambiguous prediction; higher values indicate greater decisiveness. **(c)**Brier score; bars extend downward from 0.222, the score of a non-informative model assigning every participant the cohort response rate, so bar length represents improvement over that reference. Lower Brier scores indicate better calibration. Leading unimodal and multimodal late-fusion models are shown at PreTx and PreTx + MidTx; colors follow Fig 2. All metrics derive from nested leave-one-out cross-validation.

Among unimodal models using pre-treatment biomarkers alone, TCR repertoire achieved the highest discrimination (AUROC 0.80, 95% CI 0.57–0.98; Brier 0.192), followed by FDG-PET (0.76, 0.56–0.93) and cytokines (0.54, 0.30–0.79) (Fig 2). TCR and PET exceeded the PD-L1 benchmark of 0.58, whereas baseline cytokines did not; confidence intervals were wide and overlapping throughout. Multimodal fusion improved discrimination over the best unimodal model. Late fusion of PET and TCR achieved the highest baseline AUROC (0.85, 0.65–0.99; Brier 0.175, Fig 3c), with early fusion of the same modalities performing similarly (0.81, 0.58–0.98). Across all combinations, late fusion outperformed early fusion for the same modality pairing in seven of eight comparisons, with one tie. Complete performance metrics for all models are reported in Supplementary Table 4.

#### Mid-treatment predictive performance

When mid-treatment biomarkers were incorporated, the ordering of unimodal models changed (Fig 2). Cytokines became the strongest single modality (AUROC 0.78, 0.59–0.94), driven by week-3 IL-6, while TCR performance declined (0.66, 0.42–0.91). PET performance was unchanged at 0.76, since the same baseline feature was selected. Multimodal late fusion of PET and cytokines achieved the highest discrimination overall (AUROC 0.86, 0.70–0.97; Brier 0.170, Fig 3c). All multimodal models exceeded the PD-L1 benchmark, by margins ranging from 0.12 to 0.28 AUROC. Complete performance metrics for all models are reported in Supplementary Table 5.

### Statistical Significance

The permutation null distributions were centered close to chance (mean AUROC 0.467 at PreTx and 0.481 at PreTx + MidTx), indicating that feature selection within the pipeline introduced minimal optimism (Supplementary Fig 2). Thirteen of 22 model and timepoint combinations exceeded the null at p < 0.05 (Supplementary Table 6). Unimodal TCR at PreTx achieved the lowest empirical p-value of any model (AUROC 0.796, p = 0.002), and both headline late-fusion models were significant: PET and TCR at PreTx (0.846, p = 0.008) and PET and cytokines at PreTx + MidTx (0.858, p = 0.012).

FDG-PET alone did not exceed the null at either timepoint (0.759; p = 0.078 and p = 0.096), whereas eight of the twelve fusion models containing PET did. The imaging signal was therefore not independently sufficient to discriminate response, but improved models when combined with a circulating biomarker.

Paired DeLong comparisons are reported as effect estimates rather than significance tests. Fusion improved AUROC over the best unimodal model by 0.049 (95% bootstrap CI −0.062 to 0.164) at PreTx and by 0.080 (−0.029 to 0.200) at PreTx + MidTx (Supplementary Tables 7 and 8); both intervals include zero, indicating that the incremental benefit of fusion over the strongest single modality cannot be established at this sample size. Discrimination was consistent under repeated stratified five-fold cross-validation, with a mean absolute difference of 0.029 across models and strong rank correlation between the two schemes (Spearman ρ = 0.83 at PreTx and 0.85 at PreTx + MidTx; Supplementary Table 9).

### Prediction confidence and calibration performance

Conformal prediction and calibration metrics for the leading models at PreTx and PreTx+ MidTx are summarized in Fig 3. At PreTx, unimodal TCR produced single-class predictions for 77.8% of participants, while late fusion of PET and TCR increased this to 88.9% (Fig 3b). At PreTx + MidTx, unimodal cytokines and PET with cytokine late fusion both produced single-class predictions for 85.2% of participants. Calibration improved with fusion at both settings: Brier score fell from 0.192 to 0.175 at PreTx and from 0.202 to 0.170 at PreTx + MidTx (Fig 3c). For reference, a non-informative model predicting the base response rate for every participant would achieve a Brier score of 0.222. Conformal metrics for all models are reported in Supplementary Tables 10 and 11.

Empirical coverage was 77.8% for PET + TCR late fusion at PreTx and 81.5% for PET + cytokine late fusion at PreTx + MidTx, against a target of 80% (Fig 3a). Because each participant contributes 3.7% of coverage, achievable values are quantized in steps of that size, and the small shortfall at PreTx reflects this granularity rather than systematic miscalibration.

Addition of FDG-PET to the leading blood biomarker model changed classification for a small number of individual participants, with a net gain of two participants at PreTx and one at PreTx + MidTx (Supplementary Table 12). Among participants receiving a single-class conformal prediction, classification accuracy was higher than overall accuracy at PreTx + MidTx (0.783 versus 0.704 for PET and cytokine late fusion), but not at PreTx (0.750 versus 0.778), indicating that the abstention mechanism concentrated accuracy on committed predictions inconsistently in this cohort.

### Patient-level multimodal prediction outcomes

Patient-level prediction outcomes for best-performing multimodal late-fusion models at both timepoints are shown in Supplementary Fig 3, demonstrating heterogeneous individual prediction behavior and temporal changes in classification confidence following mid-treatment biomarker integration.

### Representative patient examples demonstrating clinical utility

Representative examples from real patients illustrating multimodal prediction behavior are summarized in Table 2. These examples demonstrate distinct clinical patterns observed across patients, including resolution of ambiguous baseline predictions through multimodal integration, agreement across modalities, and temporal correction of baseline misclassification following incorporation of mid-treatment cytokine features. In addition, selected cases highlight the role of uncertainty in preventing incorrect predictions when conflicting signals are present. Together, these examples provide clinically interpretable illustrations of multimodal decision behavior observed across the cohort.

Prediction sets of {0,1} indicate valid uncertainty (0 = Responder, 1 = Non-responder). Examples illustrate four clinically relevant patterns: (1) Bidirectional resolution, in which multimodal fusion resolves ambiguity from either PET (Patient A) or biomarkers (Patient B); (2) Biomarker handoff, where mid-treatment IL-6 corrects a baseline misclassification (Patient C); and (3) Conformal safety, where conflicting signals are flagged as uncertain rather than producing an incorrect prediction (Patient D).

### Clinical decision support prototype

**Figure 4:**
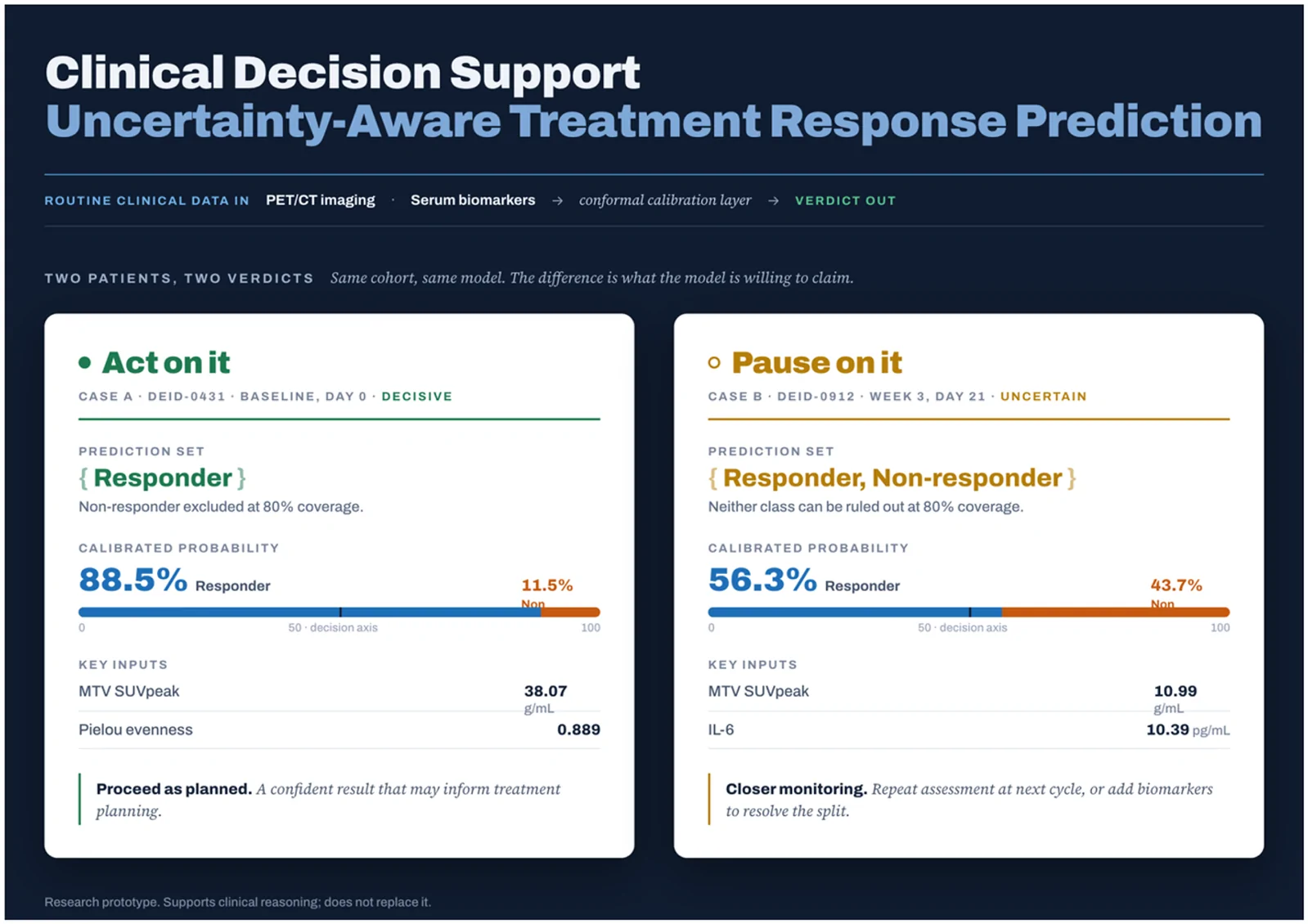
Prototype clinical decision support interface implementing the uncertainty-aware multimodal framework. Routine clinical inputs, FDG-PET/CT and serum biomarkers, are passed through modality-specific models and a conformal calibration layer to produce a patient-level output. Two contrasting cases are shown. **Case A** uses pre-treatment MTV SUVpeak and pre-treatment TCR Pielou evenness; late fusion of modality-specific probabilities yields a calibrated probability, and a single-class prediction set {responder}, in which non-response is excluded at the pre-specified 80% coverage level. **Case B** uses pre-treatment MTV SUVpeak and mid-treatment IL-6; the calibrated probability lies close to the decision boundary and the prediction set retains both classes {responder, non-responder}, indicating that neither outcome can be excluded at the same coverage level. In this situation the interface recommends closer monitoring or additional biomarker assessment rather than a change in management. The interface runs locally for privacy-sensitive use; values shown are illustrative examples.

As an early prototype, we implemented a local, browser-based clinical decision support tool using Streamlit [37] to show the clinical utility of the multimodal prediction workflow. The tool takes in baseline and mid-treatment data (consistent with our study design) and generates predictions using the multimodal framework. The interface displays model probabilities and prediction sets in a compact visualization format suitable for reporting. Fig 4 shows a pair of screens from our prototype clinical decision support interface, which demonstrates how our multimodal prediction framework might be used in a clinical setting . Example outputs illustrate baseline definitive predictions (Fig 4 Case A) and mid-treatment uncertain predictions (Fig 4 Case B). These examples highlight the ability of the interface to communicate prediction probabilities and uncertainty across clinically relevant timepoints.

## Discussion

In this study, we developed an uncertainty-aware multimodal machine learning framework for treatment response prediction in mNSCLC patients by integrating longitudinal FDG PET imaging, TCR and inflammatory cytokines through late fusion. The framework substantially outperformed PD-L1 tumor proportion score, the biomarker currently used to guide first-line treatment decisions, and provided a calibrated probability together with an explicit measure of uncertainty for every patient. To facilitate clinical translation, we implemented this model as a prototype clinical decision support interface for therapy selection and adaptation over time. To our knowledge, this study represents one of the first efforts to integrate longitudinal FDG PET imaging and circulating immune biomarkers within an uncertainty-aware multimodal framework for early treatment response prediction in mNSCLC patients.

The shift in predictive performance from baseline TCR to mid-treatment cytokines is consistent with an evolving biological response to therapy. At baseline, TCR Pielou evenness was the strongest unimodal predictor, consistent with prior studies demonstrating baseline TCR metrics influence chemoimmunotherapy response in NSCLC. TCR evenness has previously been associated with complete pathologic response in the neoadjuvant setting [38, 39], with independent cohorts corroborating the predictive value of baseline repertoire metrics across pembrolizumab- and durvalumab-based regimens [40, 41]. At mid-treatment, IL-6 was the strongest unimodal predictor consistent with prior studies demonstrating that on-treatment IL-6 correlates with response during PD-1 inhibitor therapy[42] and that elevated baseline IL-6 is associated with reduced response[43]. This temporal pattern should nonetheless be interpreted as hypothesis-generating. Given the limited sample size and the wide, overlapping confidence intervals, the apparent shift is consistent with known immunobiology but cannot be established as a reproducible finding without independent replication. Its practical implication, that the biomarkers most informative before treatment may not be those most informative during treatment, supports longitudinal rather than single-timepoint biomarker collection in future studies.

Multimodal late fusion achieved the highest discrimination at both temporal settings, and with the exception of pre-treatment cytokines, every unimodal and multimodal model exceeded the PD-L1 benchmark (Fig. 2). The incremental benefit of fusion over the strongest single modality could not be established statistically given the limited sample size, and should therefore be regarded as suggestive rather than demonstrated. The improved multimodal prediction performance likely reflects the complementary biological information captured by distinct modalities such as FDG PET imaging for metabolic activity and circulating immune biomarkers for systemic immune activity and inflammatory signaling [13, 44-46]. FDG-PET was not independently sufficient to discriminate response but contributed to most of the fusion models that exceeded the permutation null, and because PET/CT is already standard of care in NSCLC staging [47], this measurement is obtained from an existing scan at no additional cost or radiation exposure. Prior multimodal studies in NSCLC immunotherapy have similarly shown that integrating multiple data modalities improves predictive performance relative to unimodal models [17, 18]. Our findings that late-fusion outperformed early-fusion is consistent with multimodal machine learning literature[48-50], which has shown that modality-specific modeling followed by probability-level averaging often provides a robust approach for combining heterogeneous data sources. A plausible explanation is that early fusion required the relative weighting of modalities to be estimated from the training data, whereas late fusion combined modality-specific probabilities with fixed equal weights, which consume no degrees of freedom and are more robust than estimated weights when samples are small and predictors correlated. Our best multimodal AUROC of 0.86 is comparable to prior multimodal immunotherapy response prediction studies in NSCLC. *Vanguri et al*. reported an AUC of 0.80 integrating CT imaging, histopathology, and genomic features[18], while *Captier et al*. similarly demonstrated that late fusion of multimodal features improved outcome prediction [17, 18], consistent with our findings.

Direct comparisons are limited by differences in modalities, cohort size, and validation strategies. While the absolute improvement from unimodal TCR to multimodal late fusion is modest in magnitude (ΔAUROC = 0.045), the concurrent improvement in calibration (Brier score 0.192 → 0.175 at PreTx and 0.202 → 0.170 at PreTx + MidTx) and conformal efficiency (singleton rate 77.8% → 88.9%) suggests that multimodal integration provides complementary clinical value beyond discrimination alone.

Accurate calibration of predicted probabilities is fundamental to clinical safety, as overconfident predictions can adversely influence therapeutic decision-making[28]. A model intended for clinical use must convey not only what it predicts but how much confidence that prediction warrants for the individual patient, and conventional binary classifiers cannot do this: they assign a label to every patient, including those for whom the available evidence is genuinely ambiguous [51]. Conformal prediction addresses this by returning a set of plausible labels with a coverage guarantee that requires no distributional assumptions and holds at any sample size. Calibration and decisiveness are linked in this setting, since better-calibrated probabilities lie further from the decision boundary and therefore yield smaller prediction sets; the fusion models were correspondingly both better calibrated and more decisive than their unimodal counterparts (Fig. 3), consistent with prior conformal prediction studies [31, 52-54]. The clinical reading of an ambiguous set is therefore not that the patient will fail to respond, but that the measured biomarkers do not resolve the question, and such a patient warrants closer monitoring or additional biomarker assessment rather than a change in management. It is this property that distinguishes decision support from prediction, and the reason we implemented the framework as a prototype interface displaying probabilities alongside their prediction sets (Fig. 4).

Our study has several limitations. First, the analysis was conducted on a relatively small sample size from a single institution. Although nested-leave-one-out cross validation was used to mitigate overfitting and conformal prediction retains its coverage guarantee at any sample size[21], independent cohorts will be required to confirm the stability and generalizability of these findings. Moreover, the incremental benefit of multimodal fusion over the best single modality could not be established at this sample size; the reported performance gains should therefore be interpreted as exploratory. Second, external validation using independent datasets was not performed, as comparable datasets with synchronized longitudinal imaging and circulating biomarker measurements required to support this multimodal analysis were not available. Third, a prototype clinical decision support tool was developed as a proof-of-concept and was not evaluated within real-time clinical workflows. Future studies incorporating clinician feedback and workflow-based usability assessments will be needed to understand how uncertainty-aware outputs influence clinical interpretation and decision-making. Finally, variability in imaging acquisition protocols, image registration, tumor delineation, sequencing platforms, and laboratory assays across institutions may affect biomarker reproducibility, underscoring the importance of standardized acquisition and processing pipelines for broader clinical translation.

## Conclusions

We developed an uncertainty-aware multimodal framework by integrating longitudinal FDG PET imaging, TCR, and cytokines for early prediction of treatment response in metastatic NSCLC. A prototype for clinical decision support illustrates the feasibility of translating the proposed framework into clinically actionable output to support therapy selection and therapy adaptation over time. Independent validation and prospective evaluation will be essential to confirm generalizability and clinical impact.

## Supplementary Material

This is a list of supplementary files associated with this preprint. Click to download.

• SupplementaryData.docx

## Figures and Tables

**Figure 1:**
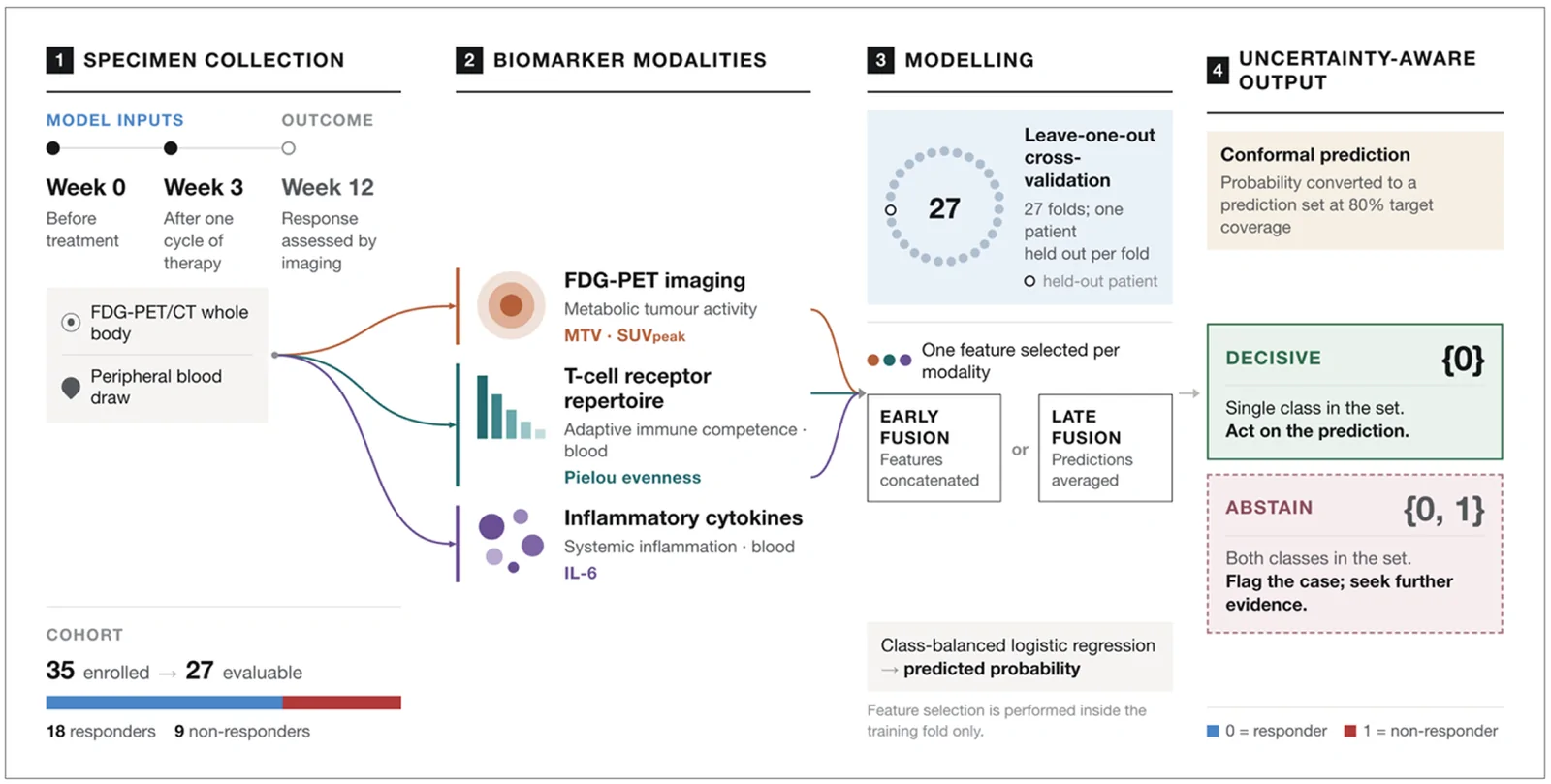
Overview of the uncertainty-aware multimodal framework for early response prediction in metastatic non-small cell lung cancer. **(1)**Participants underwent FDG-PET/CT imaging and peripheral blood sampling before treatment (week 0) and after one cycle of chemoimmunotherapy (week 3). Both timepoints served as model inputs; response was assessed independently at week 12 by RECIST v1.1 and was not used as a predictor. Of 35 participants enrolled, 27 were evaluable (18 responders, 9 nonresponders). **(2)** Three modalities were profiled, with the representative feature most frequently selected for each shown. **(3)**In each of 27 nested leave-one-out folds, one participant was held out, a single feature was selected per modality within the training set only, and modalities were combined by early or late fusion before class-balanced logistic regression. **(4)**Conformal prediction converted each probability into a prediction set at 80% target coverage. Single-class sets ({0}, responder; {1}, non-responder) represent decisive predictions; sets containing both classes ({0, 1}) indicate insufficient evidence to distinguish outcomes, flagging the participant for further assessment rather than forcing a classification. *MTV*, metabolic tumour volume; *SUV*, standardized uptake value; *IL-6*, interleukin 6.

**Table 1.** Patient characteristics (N = 35)

Characteristics	Value*
Age (years)	71 (33-84)
Sex	
Female	19 (54%)
Male	16 (46%)
Smoking history	
Former	21 (60%)
Current	7 (20%)
Never	7 (20%)
Cancer type	
Adenocarcinoma	30 (85.7%)
Squamous cell carcinoma	2 (5.7%)
Large cell carcinoma	1 (2.85%)
Other	1 (2.85%)
Missing	1(2.85%)
Stage	
IV	33 (94%)
Metastatic Recurrence	1 (3%)
Missing	1(3%)
PD-L1 tumor proportion score	
< 1%	18 (51%)
> 1%	15 (43%)
Unknown	2 (6%)
Chemotherapy regimen	
carboplatin + pemetrexed + pembrolizumab	30 (85.7%)
carboplatin + paclitaxel + pembrolizumab	5 (14.3%)
History of brain metastases	
Yes	6(17.1%)
No	20(57.1%)
Unknown	9(25.7%)
Known Driver mutation	
ALK	1 (2.9%)
BRAF	1 (2.9%)
EGFR	1 (2.9%)
ROS1	0 (0%)
Other	13 (37.1%)
None	18 (51.4%)
Missing	1(2.9%)

* Values are no. (%) or median (range)

**Table 2.** Representative patient examples demonstrating multimodal clinical utility.

Patient	True label	PET (PreTx)	PET+TCR (PreTx)	PET+Cytokines (PreTx +MidTx)	Clinical Utility
A	Non-responder (1)	{0,1} (Unsure)	{1} Correct	{1} (Correct)	**Uncertainty Resolution:** Multimodal fusion resolves PET ambiguity
B	Responder (0)	{0} (Correct)	{0} (Correct)	{0} (Correct)	**PET Resolution:** PET resolves biomarker ambiguity (TCR/Cytokines alone were unsure)
C	Responder (0)	{0,1} (Unsure)	{1} Incorrect	{0} (Correct)	**Temporal Handoff:** MidTx IL-6 rescues PreTx error
D	Responder (0)	{1} (Incorrect)	{0,1} (Unsure)	{0,1} (Unsure)	**Safety:** Fusion flags ambiguity where PET makes an error

## Abbreviations

AUROC: area under the receiver operating characteristic curve
CDS: clinical decision support
CDSS: clinical decision support system
chemoICI: chemotherapy combined with immune checkpoint inhibitor
FDG: 18F-fluorodeoxyglucose
IL-6: interleukin 6
LOOCV: leave-one-out cross-validation
MidTx: after one cycle of chemoimmunotherapy
mNSCLC: metastatic non-small cell lung cancer
MTV: metabolic tumor volume
PD-L1: programmed death ligand 1
PET: positron emission tomography
PreTx: before treatment
RECIST: Response Evaluation Criteria in Solid Tumors
SUV: standardized uptake value
TCR: T-cell receptor
TLG: total lesion glycolysis
TMB: tumor mutation burden
